# PATHOGEN APATHY RESULTING FROM SKN-1 ACTIVATION: A MARK OF RESILIENCE OR A RATTLED GUT FEELING?

**DOI:** 10.1093/geroni/igad104.3514

**Published:** 2023-12-21

**Authors:** Tripti Nair, Brandy Weathers, Nicole Stuhr, James Nhan, Vandita Gorla, Sean Curran

**Affiliations:** University of Southern California, Los Angeles, California, United States; University of Southern California, Los Angeles, California, United States; University of Southern California, Los Angeles, California, United States; University of Southern California, Los Angeles, California, United States; University of Southern California, Los Angeles, California, United States; University of Southern California, Los Angeles, California, United States

## Abstract

Innate immunity is a key driver in promoting survival against xenobiotic stressors and hostile environments and an appropriate immune response is essential to organismal health across lifespan. Caenorhabditis elegans encounter pathogens in their natural environment and initiate cytoprotective and immune responses that can have long-lasting effects on health, even after the pathogen has been neutralized. Pseudomonas aeruginosa (PA14) is an opportunistic pathogen that is lethal to C. elegans with prolonged exposure. C. elegans employ multiple defense mechanisms to ensure survival upon exposure to pathogens to defend against the current infection and to avoid continued exposure, including activation of the key cytoprotective transcription factor SKN-1 (mammalian homologue Nrf2). Although wild type C. elegans quickly learn to avoid pathogens, our current work documents a peculiar apathy to pathogen in animals with constitutive activation of SKN-1. The outcome is surprising as the role of SKN-1 is to protect an animal from intracellular and environmental insults. This behavior is mediated by tissue specific actions of SKN-1 that initiate cell non-autonomous responses for pathogen avoidance, survival, and metabolic adaptation. Based on transcriptional signature of animals with SKN-1 activation upon exposure to PA14 we observed that the apathy behavior is independent of somatic lipid depletion, thus uncoupling these two exceptional physiological responses. Further, we define the role of neurosignalling molecules in the gut-brain axis that drive the apathy response to pathogens. Our work reveals new insights into how animals perceive pathogens in the environment and subsequently alter behavior and cellular programs to promote survival.

